# Supplementary material for: Are carbon emissions trading and green financial instruments synergistic? -Comprehensive quantitative research based on content analysis
Source: PLoS One. 2024 Mar 7;19(3):e0298601. doi: 10.1371/journal.pone.0298601 (PMC10919601; doi:10.1371/journal.pone.0298601)
Supplement: S1 Data — (ZIP) [file pone.0298601.s001.zip › Data/地级市Z_F工作报告.zip!/╔╜╢1⁄2/║╩╘≤╩╨/└┤╫╘╣1⁄2╓┌║┼í╛┬φ┐╦╩2╛▌═°í┐_2012.docx]

各位代表:

　　我代表市人民政府向大会报告工作，请予审议，并请市政协委员提出意见。

　　一、过去四年的工作回顾

　　2008年市十七届人大一次会议以来，全市人民在市委的坚强领导下，认真贯彻落实科学发展观和省委、省政府决策部署，紧紧围绕“科学发展、和谐发展、跨越发展、更好更快发展”总体思路，牢牢坚持“高境界、高标准、高效率、高效益”工作要求，大力实施工业化、城镇化“双轮驱动”战略，全力加快“五大基地一大产业”建设，着力保障和改善民生，经济社会发展取得了令人瞩目的成就。

　　——经济综合实力明显提升。地区生产总值由2007年的694亿元增加到2011年的1475亿元，年均增长14.6%。地方财政收入由42.1亿元增加到111.5亿元，年均增长27.6%，总量在全省前移2个位次，列周边9市第2位。各项存款余额由570亿元增加到1326亿元，贷款余额由444亿元增加到877亿元，年均分别增长23.5%和18.5%。主要经济指标增幅均居省内及周边9市前列。

　　——转方式调结构取得明显进展。三次产业比由26.2∶47.7∶26.1调整到15.5∶53.7∶30.8，二三产业比重提高10.7个百分点。产业集群化发展态势日益凸显。高新技术产业产值占规模以上工业的比重提高5.6个百分点。省下达的节能减排任务全面完成。

　　——城乡面貌明显改观。城镇规划建设管理快速推进，承载功能大幅提升。城镇化率达到37.5 %，提高7.9个百分点。创建成为中国优秀旅游城市和省级园林城市。济菏高速、德商高速南段建成通车，东明至巨野、曹县至鄄城一级公路完成升级改造，改建国省干线公路640公里，新建改造农村公路4490公里。新农村建设扎实推进。城乡环境综合整治收到明显成效。

　　——人民群众生活明显改善。教育、科技、文化、卫生、体育等各项社会事业全面发展，城镇就业和农村劳动力转移就业较快增加，社会保障体系逐步健全。城镇居民人均可支配收入由9715元增加到16658元，农民人均纯收入由4023元增加到7119元，年均分别增长14.4%和15.3%；城乡居民消费水平有较大提高。

　　各位代表，过去的四年，是我市经济社会发展最好最快的四年，是城乡面貌变化最大的四年，是人民群众得到实惠最多的四年，也是为打造区域科学发展高地奠定基础、积蓄力量的四年。为此，全市上下群情振奋，倍受鼓舞，感到无比骄傲和自豪！

　　四年来，我们主要做了以下工作：

　　（一）大力推进招商引资和大项目建设，经济发展后劲进一步增强。建立市县领导分包基地、产业和重点项目责任制，实行市级领导带队集中招商、小分队招商、产业招商、以商招商等多种招商方式，完善大项目建设月调度、季观摩、年终考核机制，招商引资和大项目建设取得显著成效。东明石化120万吨高硫重油综合利用、玉皇化工20万吨顺丁橡胶、步长制药、巨润新型建材等28个投资10亿元以上的大项目竣工投产；全市煤炭产量达到1250万吨，龙堌、赵楼、郭屯煤矿投产运营；菏泽电厂三期、华润电厂一期并网发电；建成平原水库3座，洙水河航道具备通航条件，为经济发展提供了有力支撑。

　　（二）大力推进“1655”企业成长计划，工业经济质量效益显著提高。加强运行调度，积极搞好服务，及时帮助企业解决生产经营中的困难和问题，加快了企业膨胀步伐。规模以上工业企业发展到2119家，增加814家；主营业务收入由906亿元增加到3397亿元，利税由69亿元增加到457亿元，年均分别增长39.2%和60.4%。60户重点企业完成主营业务收入1004亿元、实现利税157亿元，年均分别增长32.6%和51.2%。主营业务收入过100亿元的企业达到2家，过10亿元的32家，东明石化进入中国500强。6家企业在海外成功上市。深入实施“质量兴市”战略，创山东名牌产品45个、山东省著名商标34个、中国驰名商标4个，省长质量奖1个，企业整体素质明显提高。

　　（三）大力推进城乡建设，城镇化进程明显加快。高水平编制城市规划，市区和县城控制性详规覆盖率均达到100%。市区新建改造南外环路、长江路、黄河东路等道路140余条，新建长城路桥等桥梁18座；铺设污水管网289公里、雨水管网436公里，碧水工程取得初步成效；完成菏泽大剧院、演武楼、赵王河综合治理、曹州牡丹园提升改造等重点工程96项；新增绿化面积530万平方米，绿化覆盖率达到40%。8个县的县城建设全面加快，部分县城面貌发生巨大变化。大力实施城市综合开发，全市竣工房地产面积2207万平方米，其中商业面积286万平方米。扎实推进农村住房建设和危房改造，启动整村改造项目567个，完成农房建设17.7万户，改造危房10.3万户，城乡居民生活条件明显改善。

　　（四）大力推进农副产品生产加工基地建设，农村经济全面发展。粮食总产达到117亿斤，连续八年增产；经济作物面积发展到702万亩；经济林面积达到47.5万亩，森林覆盖率达到33.6%；畜禽养殖小区发展到1860个，出栏量达到1.2亿头（只）；水产品养殖效益有较大提高。加大农业科技推广力度，科技对农业增长的贡献率达到50%。农村二、三产业加速发展，规模以上农副产品加工企业发展到1249家，农民专业合作组织2759家。农业标准化基地面积发展到280万亩，增加160万亩。连年开展农田水利建设大会战，新增有效灌溉面积157万亩。农作物耕种收机械化率达到80%。

　　（五）大力推进科技创新、节能减排和节约集约用地，科学发展水平明显提高。全社会研发投入累计完成30.5亿元，取得重要科技成果789项，新增省级以上企业技术中心16家，市级以上高新技术企业达到161家，其中省级12家、国家级3家，全市高新技术产业产值达到799亿元，年均增长57.6%。建立健全节能预警调控机制，深入开展能效对标活动，实施节能技改项目260个，关停落后产能企业350家，能耗水平明显下降。不断加大环境保护力度，134家市控以上企业全面完成升级改造，市县乡34处污水处理厂全部建成投用，三条主要河流出境断面水质持续改善。2008年代表山东省接受迎淮核查取得第一名，被省政府评为“十一五”时期总量减排目标考核突出贡献单位和生态建设先进市。坚持节约集约利用土地，挖潜盘活土地4万亩，新开发整理耕地11万亩，连续多年实现耕地总量动态平衡。

　　（六）大力推进“四大载体”建设，服务业发展步伐加快。以推进重点城区、重点园区、重点企业、重点项目“四大载体”为着力点，创新发展思路，加大开发力度，优化消费环境，促进了商贸物流、文化旅游业快速发展，服务业增加值达到454.6亿元, 年均增长16%。牡丹商贸城、单县义乌小商品城、巨润建材物流园等86个服务业大项目相继建成投用，银座、华瑞、三信等企业的辐射带动能力日益增强。社会消费品零售总额达到775亿元，年均增长19.9%；进出口总额达到27.4亿美元，年均增长33.9%。文化产业增加值达到41.5亿元，年均增长25.6%；旅游总收入达到46亿元，年均增长32.6%。

　　（七）大力推进民生建设，社会大局和谐稳定。坚持每年办好十件民生实事，较好地解决了事关群众切身利益的实际问题。累计新增城镇就业36.8万人、农村劳动力转移就业80万人。新农保、城镇居民养老保险快速推进，实现全覆盖。城乡低保、大病救助、农村五保供养标准大幅提高。新增集中供热覆盖面积2100万平方米、民用天然气用户20万户，新增公交线路41条、公交车386辆，新增农村饮水安全人口158.6万人。建成投用保障性住房26340套，解决了7.9万中低收入群众的住房困难。维修改造中小学校舍307万平方米，新增幼儿园205所，新建城区学校88处，公开招聘教师5617名，办学条件明显改善。医药卫生体制改革不断深化，基层医疗卫生机构综合改革全面完成，新农合财政人均补助由40元增加到200元。放映农村公益电影30万场次，新增有线电视用户38万户；新建乡镇综合文化站147个、农村文化大院2617家，入选国家级、省级非物质文化遗产名录项目数量均居全省第一，元代沉船和定陶汉墓考古发掘取得重大成果。成功承办第十一届全运会武术散打比赛，被国家体育总局授予重大贡献奖。加大违法生育查处力度，建立健全计划生育利益导向机制，人口自然增长率下降到4.97‰。食品安全综合治理机制基本建立，食品药品监管力度不断加大，保障了人民群众饮食用药安全。强化基层基础工作，狠抓隐患排查治理，安全生产形势持续好转。深化“平安菏泽”建设，社会治安形势持续稳定。对口援川任务提前一年完成。民族宗教、对台事务、外事侨务、妇女儿童、民兵预备役、统计、物价、人防、气象、地震、史志、老龄、残疾人、关心下一代等各项事业都有了较快发展。

　　（八）大力推进政府自身建设，行政效能进一步提高。自觉接受人大法律监督、工作监督和政协民主监督，认真办理人大代表议案、建议和政协委员提案，办复率达到100%。积极推进行政审批制度改革，精简审批事项78项，服务质量和办事效率不断提高。全面实行服务承诺、限时办结、部门主要负责人电话公开制度，充分发挥市长公开电话、行风热线等便民渠道作用，及时解决了一大批群众关心的热点难点问题。认真落实党风廉政建设责任制，强化行政监察和审计监督，政府系统廉政建设取得新成效。

　　以上工作成绩的取得，是市委科学决策、正确领导的结果，是市人大、市政协和社会各界有效监督、大力支持的结果，是全市广大干部群众奋力拼搏、扎实苦干的结果。在此，我代表市人民政府，向全市人民，向各民主党派、工商联、人民团体和社会各界人士，向驻菏部队、武警官兵和中央、省驻菏单位，向所有关心支持菏泽发展的朋友们，表示崇高的敬意和诚挚的感谢!

　　同时，我们也清醒地认识到，我市发展中仍然存在不少困难和问题。主要是：经济总量小，人均水平低；大项目少，工业化、城镇化水平低；自主创新能力弱，对外开放程度不高；铁路、公路等重大基础设施建设相对滞后，社会事业欠账较多；政府破解发展难题的能力和依法行政水平有待进一步提高等等。对此，我们一定高度重视，采取有力措施，切实加以解决。

　　二、今后五年的总体要求和奋斗目标

　　新一届政府履职的五年，是我市加快推进工业化、城镇化的重要机遇期，也是转方式、调结构的攻坚期，更是打造区域科学发展高地的关键期。虽然我们面临着错综复杂的国内外经济形势和日趋激烈的区域竞争，但更面临着千载难逢的发展机遇。省委、省政府作出支持菏泽打造鲁苏豫皖交界地区科学发展高地的重大战略决策和部署，专门出台了进一步支持菏泽加快科学发展的20条政策措施，支持力度之大，政策含金量之高，前所未有。我市所属的中原经济区发展规划上升为国家战略，也为我们争取国家支持提供了有利条件。从我市看，经过多年的发展和积累，煤炭开发全面加速、深入推进，日东输油管道基本建成，大项目、大企业支撑作用日益突出，煤电化工、石油化工等产业集群优势初步显现，可持续发展能力不断增强。全市上下人心思干，人心思进，凝心聚力、干事创业的热情空前高涨。刚刚胜利闭幕的市十二次党代会，为我们绘就了今后五年的美好蓝图。只要我们坚定信心、振奋精神，抓住机遇、乘势而上，就一定能够战胜任何困难挑战，就一定能够跨越赶超、后来居上，就一定能够实现打造区域科学发展高地的宏伟目标！

　　今后五年，我市经济社会发展的总体要求是：以邓小平理论和“三个代表”重要思想为指导，深入贯彻落实科学发展观，认真落实市十二次党代会精神，坚持“四高”要求和“六个必须”，牢牢把握主题主线，紧紧抓住工业化、城镇化、农业现代化和繁荣发展服务业四项工作重点，加快培育“五大基地一大产业”，着力深化改革开放，着力加强科技创新，着力推进节能减排，着力保障和改善民生，努力推动全市科学发展、和谐发展、跨越发展、更好更快发展，初步形成鲁苏豫皖交界地区的产业发展高地、自主创新高地、城乡统筹发展高地、对外开放高地、生态发展高地和社会事业发展高地。

　　全市经济社会发展的主要目标任务是：

　　一是坚持加快发展不动摇，不断增强经济综合实力。加快发展是我市摆脱欠发达面貌、打造区域科学发展高地的关键所在，更是各级政府的首要责任、首要任务、首要工作。必须始终坚持聚精会神搞建设，一心一意谋发展，千方百计膨胀经济总量，提升发展质量，增强综合实力，全力推动经济社会跨越发展。到2016年，地区生产总值达到2600亿元以上，年均增长13％；地方财政收入达到260亿元以上，年均增长20％。总量跨入全省中等行列，人均指标在全省实现位次前移，居周边地区前列。

　　二是坚持加快转变发展方式不动摇，全力推动科学发展。科学发展是时代的要求，也是加快发展的根本保证。必须把加快转变发展方式作为经济工作的主动追求和自觉行动，切实加强科技创新，大力推进资源节约型、环境友好型社会建设。全社会研发投入达到41.6亿元，年均增长29.6%。高新技术产业产值达到3500亿元，年均增长36%。市级以上高新技术企业达到280家，省级以上企业技术中心55家、国家级6家。全面完成节能减排任务目标，森林覆盖率达到36%，生态建设在周边地区保持领先水平。

　　三是坚持调整经济结构不动摇，大力促进产业优化升级。优化提升一产、做大做强二产、加快发展三产，三次产业比调整为9∶55∶36，二三产业比重提高6.5个百分点。积极发展现代农业，确保粮食产量稳定增长，经济作物面积增加200万亩。国家级农业产业化龙头企业达到4家、省级50家。加快发展服务业，重点抓好大型物流园区、专业批发市场、城市综合体和旅游景区建设，推动商贸物流、文化旅游业跨越发展，努力把文化旅游业培植成为新兴支柱产业。服务业增加值达到1000亿元，占地区生产总值的比重提高5个百分点以上。实现社会消费品零售总额1700亿元，年均增长17%；旅游总收入105亿元，年均增长18%以上。着力培育“五大基地一大产业”，打造百亿级企业、千亿级产业。战略性新兴产业、石油化工、煤电化工、农副产品生产加工基地主营业务收入分别达到2800亿元、2400亿元、1500亿元和2000亿元。规模以上工业企业实现主营业务收入9000亿元以上，年均增长22%以上，过1000亿元的企业1家、过500亿元的2家、过100亿元的20家以上，在工业产业规模及优化升级上领跑周边地区。

　　四是坚持加快城镇化进程不动摇，着力完善提升城镇承载功能。城镇是经济发展的重要载体，城镇化是现代化建设的必由之路。围绕建设鲁苏豫皖交界地区中心城市目标，不断提高城市规划建设管理水平。城镇化率达到47.5%，年均提高2个百分点。市区建成区面积达到120平方公里，人口规模达到100万人以上；40万人以上的县城发展到3个，建成3万人以上的中心镇24个、2万人以上的小城镇55个；每年重点打造100个生态文明村。加快公路、铁路、水利、电力、通讯等基础设施建设，为打造区域科学发展高地提供强力支撑。

　　五是坚持改革开放不动摇，进一步增强发展活力。改革开放是推动跨越发展、打造区域科学发展高地的强大动力。积极推进重点领域和关键环节改革，健全完善国有资产监管体制，不断深化农村各项改革。加大政策支持力度，放宽准入领域，营造良好环境，促进民营经济和小微企业快速健康发展。大力实施对外开放战略，以更大气魄、更大决心、更大力度推动对外开放，提高开放的层次和水平，更多地利用外部资源和要素。抓住产业转移大好机遇，瞄准世界500强、国内500强、中央和省内大企业，面向港澳台、长三角、珠三角等重点区域，加大招商力度，努力引进一批龙头型、旗舰型、领军型项目。进出口总额达到55亿美元，年均增长15%；利用外资实现重大突破。

　　六是坚持区域协调发展不动摇，加快发展县域经济。县域经济在我市经济发展中具有举足轻重的战略地位。县域兴则全市兴，县域强则全市强。必须把发展县域经济放到更加重要的位置，鼓励各县区立足实际，发挥比较优势，选准突破口和着力点，大力发展特色经济，培育产业集群，壮大经济实力。支持定陶、鄄城、成武加快发展，促进县域经济协调发展。到2016年，各县区地方财政收入全部超过15亿元，其中过30亿元的4个，过50亿元的1个。坚持产业兴镇，提升城镇功能，加快发展壮大镇域经济，培育一批新的经济增长点。

　　七是坚持民生优先不动摇，努力建设幸福菏泽。民生连民心，民生大如天。认真落实民生政策，努力加大财政投入，千方百计扩大就业，健全社会保障体系，加快发展社会事业，不断增加群众收入。城镇居民人均可支配收入、农民人均纯收入年均分别增长12%以上。每年集中办好一批事关群众切身利益的实事好事，使人民生活水平和质量有一个大的提高，让全市人民都能享受到改革发展的成果，生活得更加殷实、更有尊严、更加幸福！

　　三、2012年的目标任务和工作措施

　　五年发展看头年。做好今年的政府工作，对于实现打造区域科学发展高地目标至关重要。全市经济社会发展的主要预期目标是：地区生产总值增长13%以上；地方财政收入增长20%；规模以上工业增加值增长20%以上；规模以上固定资产投资增长20%以上；社会消费品零售总额增长17%；进出口总额增长15%以上；城镇居民人均可支配收入和农民人均纯收入均增长12%以上；居民消费价格涨幅控制在4%以内；城镇登记失业率控制在3.8%以内；人口自然增长率控制在6.8‰以内；完成省下达的节能减排任务。

　　围绕实现上述目标，重点抓好以下十项工作：

　　（一）加快推进工业化进程。坚持把推进新型工业化作为打造区域科学发展高地的重大任务，奋力攻坚，重点突破。一是全力抓好大项目建设。围绕建设“五大基地一大产业”，围绕打造百亿级企业、千亿级产业，高水平策划储备一批重大项目，储备生产性项目投资额400亿元以上，列入省重点的项目个数继续保持全省领先。严格落实市县领导干部包保责任制，强化大项目建设推进机制，确保万福煤矿、金正大硝基肥、浮龙湖风力发电等50个大项目开工建设，确保东明石化重质油综合利用、华盛荣镁合金、洪业化工己内酰胺等50个大项目竣工投产。完成固定资产投资660亿元。二是推动企业快速膨胀。深入实施“1655”企业成长计划，着力培育壮大60户重点企业，加强企业家队伍建设，引导推动企业加快重组合作，拓宽投融资渠道，抓技改、上项目，抓管理、上水平，实现快速滚动发展，尽快做大做强。确保“1655”企业实现主营业务收入2800亿元，增长35%；60户重点企业主营业务收入1360亿元，增长35%。其中，主营业务收入过100亿元的企业4家，过10亿元的40家，争创山东名牌产品5个以上、山东省著名商标15个以上，申请发明专利560件。三是加快产业转型升级。加大化工、纺织、食品等传统产业技改力度，着力抓好玉皇化工异丁烷脱氢、湖西王汽车零部件产能扩建等100个技改项目建设，完成技改投入200亿元，增长20%。加大战略性新兴产业培育力度，重点围绕发展新医药、新材料、新能源和机电设备制造业，着力抓好东药雄烯二酮、达驰电气高效节能变压器等100个项目建设，战略性新兴产业实现主营业务收入1200亿元，增长30%。加大高新技术重点项目建设力度，推动高新技术企业快速发展，确保市级以上高新技术企业发展到200家，高新技术产业产值达到1100亿元，增长40%，占规模以上工业总产值的比重提高2个百分点以上。四是提升园区发展水平。进一步加强园区配套设施建设，健全商住、物流、金融等服务体系，提升园区功能。高标准制定产业集群发展规划，明确产业定位，推进产业集聚集约发展，着力打造一批产业特色突出、规模优势明显的专业化园区。力争市高新区通过省政府验收，支持市开发区申报国家级开发区。

　　（二）加快推进城镇化进程。进一步加大城镇建设力度，加快推进城乡统筹发展，确保城镇化率提高2.5个百分点。一是高起点规划。注重规划衔接，突出地方特色，精心搞好市区总体规划修编和重点工程设计，修订完善各类专项规划；加快县城总体规划修编，统筹做好乡镇和村庄规划编制。严格规划管理，维护规划的权威性和严肃性。二是高标准建设。切实增强质量意识、精品意识，力争把每一项工程都建成精品。市区完成胜利广场等重点工程建设，开工建设规划展览馆、赵王河下游治理等40余项公共建设工程，完善提升碧水工程，搞好黄河路、人民北路等道路污水管网建设，新增绿化面积300万平方米，争创国家级园林城市。加快县城和重点镇基础设施建设，不断完善公共服务功能，增强综合承载能力，吸引有条件的农民向城镇聚集。加快建设德商高速（菏泽至鄄城段）、东新高速（东明段）、机场路，开工建设丰东路（定陶至济宁界）、220国道（郓城至菏泽段）、济董路（鄄城至郓城段）升级改造工程，积极实施电网建设“7511”工程。三是高效益开发。加快城市综合开发和“城中村”改造，重点抓好一批规模大、带动能力强的片区开发项目，高标准搞好配套建设，努力打造精品楼盘。全市开工建设房地产面积1770万平方米、竣工1300万平方米，其中市区开工770万平方米、竣工400万平方米。新建农房4万户、改造危房1万户。四是高水平管理。进一步完善城市管理机制，加快推进数字化城市管理，重点加强市政、市容、绿化、交通管理。深入开展城乡环境综合整治，认真解决集市占道经营、城区乱搭乱建等问题，努力把我们的家园打扮得更加整洁优美、更加清新靓丽。

　　（三）加快发展服务业。以“四大载体”建设为抓手，用好引导资金，加大政策扶持，加快发展商贸物流、文化旅游业，服务业增加值达到540亿元，增长15%。一是加快发展商贸流通业。积极开展“十百千”企业和商业示范社区创建，着力打造牡丹商业步行街、麟州商业街、舜师步行街等一批特色商业街区。搞好“农超对接”、“区超对接”和农产品现代流通综合试点，积极构建现代农村消费流通网络。着力推进牡丹商贸城西片区、舜王国际药城二期、万隆国际批发城等十大专业批发市场建设，专业批发市场实现交易额1100亿元，增长22%。二是加快发展现代物流业。围绕重点产业发展，抓好重要资源、大宗货物集散，着力推进巨野港口、东明石油化工、菏泽（鄄城）物流园等十大物流园区建设，力争年交易额过100亿元的物流园区达到4个。三是加快发展文化产业。深入发掘“一城四乡”文化潜力，高水平策划论证项目，加大资金投入，着力抓好巨野农民书画、鄄城鲁锦等七大文化产业基地建设，着力抓好鲁西南民俗文化、东明庄子文化等十一大文化园区建设，推动文化产业快速发展。四是加快发展旅游业。以打造特色旅游目的地为目标，突出抓好曹州牡丹园、孙膑旅游城、水浒好汉城、浮龙湖等十大景区的完善提升，精心搞好旅游产品组合包装和旅游线路策划设计，加大宣传推介力度，广泛营造声势，高水平办好菏泽国际牡丹花会，大力提高花会综合效益和影响力。实现旅游总收入65亿元，增长41%。同时，积极办好中国林产品交易会、中国（菏泽）农资交易会等展会，促进会展经济较快发展。

　　（四）加快发展现代农业。一是进一步调整优化农业结构。全面落实国家强农惠农富农政策，认真组织实施国家新增千亿斤粮食产能规划，大力开展粮食高产创建活动，粮食作物面积稳定在1500万亩左右、总产120亿斤以上，实现“九连增”。深入实施蔬菜、畜牧、苗木花卉、果业、渔业五大产业振兴规划，加快油用牡丹、山药、芦笋、中药材等特色产业发展，经济作物面积达到750万亩。新增造林面积15万亩。扎实推进标准化示范场区建设，畜禽出栏量达到1.8亿头（只）。搞好黄河故道高效生态渔业综合开发。二是着力推进农业产业化经营。重点抓好52家支撑企业，促进规模快速膨胀，力争新培育市级以上农业产业化龙头企业30家，其中国家级1家、省级8家，新增农民专业合作社200家。加强农业标准化生产基地建设，新增基地面积20万亩；积极打造农业品牌，新增无公害农产品、绿色食品、有机食品“三品”认证40个。三是努力提高农业科技水平。加大投入强度和工作力度，加强农技推广服务体系建设，大力培训农村实用人才，积极推广配方施肥等先进实用技术，进一步提高农业综合生产能力。做好动植物疫病防控工作，确保不发生大的病虫灾害。四是大力改善农业生产条件。完善多元化投入机制，强化以水利为重点的农村基础设施建设。继续实施农田水利会战，精心组织好小农水重点县和现代农业小麦产业项目县建设，新增有效灌溉面积60万亩。积极推广农机节本增效技术，农作物耕种收机械化率达到82%以上。

　　（五）加快对外开放步伐。对外开放是解放思想、更新观念的必然要求，是提升产业层次、增强发展实力的必然选择。按照打造区域对外开放高地要求，放宽视野，提升境界，强化措施，全面提高对外开放的层次和水平。一是狠抓招商引资。着力优化投资环境，营造亲商、安商、富商的良好氛围。不断创新招商方式，提高招商实效，积极参加香港山东周、台湾山东周、西洽会等重大招商活动，办好全市春秋两次投洽会。对重大项目，主要领导亲自谈、具体抓，努力在招大引强上实现新突破，每个县区引进3-5个投资10亿元以上的大项目。二是加快发展对外贸易。充分发挥中国木草柳出口基地、东明精细化工出口基地、巨野农产品质量安全示范区的带动作用，扩大果蔬、木制品、服装等优势产品出口，提高高新技术产品、机电产品出口比重；落实鼓励进口政策，加大原油等紧缺资源、先进技术、关键设备进口，实现进出口总额31.5亿美元。尽快启动菏泽口岸和保税区物流基地建设，改善对外开放的大环境。三是加强对外交流合作。鼓励有实力的企业赴海外投资、承包工程和建立生产基地，推进劳务合作和经济技术交流。进一步提高友城合作水平。

　　（六）加快转变经济发展方式。一是着力加强科技创新。大力推进产学研合作，加快国家创新药物孵化基地等创新平台建设，建立院士工作站3家。引进专业技术和管理人才870名，其中博士60名以上。健全多元化投融资机制，确保全社会研发投入占地区生产总值的比重提高到0.9%。继续大力实施技术创新提升工程，管好用好专项资金，突出抓好10项技术创新重大专项、10个重点产学研合作项目，争取列入省级以上科技计划35项；取得重要科技成果100项。二是扎实推进节能降耗。进一步完善节能预警调控机制，全面开展节能审查监察。着力抓好工业节能，把能效对标范围扩大到年耗能500吨标煤以上企业，淘汰改造高耗能设备1500台套，实施循环经济项目30个，突出抓好方明化工160吨锅炉改造等100个节能项目建设。同时，扎实推进交通、建筑、公共机构等重点领域节能，着力降低能耗水平。三是加大环境保护力度。严格落实新上项目环评和“三同时”制度，加大污染治理力度，加强环境执法监管，确保三条主要河流水质稳定达到三类标准，大气环境质量明显改善。认真抓好东明、鄄城农村环境连片整治示范工程，启动建设2处市级以上自然保护区。全面做好迎淮核查准备工作，确保取得优异成绩。四是大力提高节约集约用地水平。进一步增强依法用地意识，对违法用地行为严肃进行责任追究。严格建设用地管理，认真执行投资强度、容积率等控制性标准，全面清理闲置和低效利用土地。切实加强农村宅基地管理。统筹安排城乡建设用地增减挂钩，努力拓展用地空间，保障用地需求。

　　（七）加强财税金融工作。财政是经济发展的“晴雨表”，也是经济发展的“调节器”。依法加强税收征管，确保应收尽收。加大监督检查力度，强化县乡收入管理，坚决杜绝虚收空转，切实提高财政收入质量。优化支出结构，按照保民生、保运转、保发展要求，加大对社会事业、自主创新、节能减排、战略性新兴产业等方面的支持力度，积极扶持税收贡献大、地方收益多的企业加快发展，不断壮大地方财源。强化预算约束，严格支出管理，大力压缩一般性开支。切实加强政府债务管理，严格控制债务规模，切实防范和化解政府债务风险。

　　金融是现代经济的核心，金融活则全盘活。打造区域科学发展高地，金融必须先行。进一步优化金融生态环境，积极搭建银企合作平台，鼓励金融机构加大对项目建设和企业运行的信贷支持，力争新增贷款150亿元以上。大力引进市外金融机构，新设立国有商业银行1家、股份制商业银行2家、村镇银行3家。加快推进企业上市，新增上市企业4家，拓宽直接融资渠道，提升融资规模和水平。

　　（八）加强民生建设。怀着对人民群众的深厚感情和强烈的事业心、责任感，扎实做好各项民生工作。今年，集中力量办好十件实事。一是努力扩大就业。新增城镇就业9万人，农村劳动力转移就业16万人。二是提高社会保障水平。新农合、城镇居民医保财政补助标准均提高40元。城乡低保标准分别提高每人每月40元和每人每年400元，城乡大病救助标准均提高500元，农村“五保”供养标准提高400元。三是加快保障性住房建设。开工建设保障性住房12387套，建成6000套。四是改善办学条件。新建改造中小学校舍50万平方米，新增幼儿园200所，新建城区中小学15所，公开招录中小学教师1500名；完成菏泽学院二期工程，加快市职业技术学院建设进度，做好菏泽艺校改扩建和市体校新校区前期工作。五是改善医疗卫生条件。在省统一规划的2349所村卫生室，全部实行国家基本药物零差价销售。启用市中医院病房楼，开工建设市立医院、市二院扩建工程，做好市妇幼保健院迁建前期工作。六是繁荣文化事业。完成市图书馆建设，开工建设市艺术馆、博物馆，做好市科技馆、全民健身中心和老年大学建设前期工作。各县区文化馆、图书馆力争全部建成，每个县城至少建成1处数字影院，新建农村文化大院400家。送戏下乡1200场、送电影下乡7万场。实现有线电视村村通，发展数字电视用户40万户。七是加强计划生育利益引导。及时足额兑现计划生育家庭奖励资金，为农村所有独生子女和合法生育双女绝育家庭免费办理新农合，为所有计划生育贫困家庭大学新生提供资助。八是实施农村饮水安全工程。新增农村饮水安全人口40万人。九是改善生活环境条件。市区新增集中供热覆盖面积1000万平方米、民用天然气用户2万户，每县区至少新建1-2处乡镇垃圾中转站和污水处理厂。十是改善交通条件。开工建设菏泽火车站提升改造工程。新建改造市区道路15条、农村公路600公里；新建市区桥梁3座，改造农村窄危桥30座。市区新增公交线路6条，开通公交车的县城达到7个。

　　（九）加强和创新社会管理。进一步健全安全生产责任体系，加大对煤矿、化工等重点行业和人员密集场所的监管，及时排查消除各种隐患，确保安全生产事故起数、死亡人数、受伤人数和经济损失全面下降，坚决遏制重特大安全事故发生。加强消防基础设施、装备和专职队伍建设，提高社会抵御火灾的能力。加大食品安全专项整治力度，深入实施食品安全示范创建和食品药品安全放心工程，加快食品药品检验中心建设，着力完善监管长效机制，切实保障人民群众饮食用药安全。不断加强市场监管，严厉打击各种制售假冒伪劣商品行为。进一步强化信访工作，妥善化解各类矛盾纠纷。健全社会风险防范、突发事件应急机制，努力提高预防预警和应急能力。强化社会治安综合治理，深入开展严打整治活动，加强对流动人口、特殊人群的管理服务，强化边界、城乡结合部等重点区域治理，努力打造平安建设高地。

　　（十）加强政府自身建设。为人民服务是政府的天职，让群众满意是政府工作的最高追求。我们将牢记宗旨，不辱使命，不负重托，努力打造为民、务实、清廉、高效的政府。一是认真开展“落实年”活动。政府最重要的工作就是抓落实。今年，将在全市政府系统组织开展“落实年”活动，着力提高政府执行力和行政效能，促进干部作风转变。全面建立目标责任制，层层分解目标、落实责任，严格实行行政问责。加强政务督查和效能监察，确保政府工作政令畅通、令行禁止、高效运行，确保政府确定的目标、制定的政策、部署的工作都能够一抓到底，取得群众满意的结果。二是提高依法行政水平。把依法行政贯穿于政府工作的各个环节，严格按照法定权限、法定程序行使权力、履行职责。坚持重大事项集体决策、专家咨询、社会公示与听证、决策评估等各项制度，努力提高政府决策民主化、科学化水平。继续深化行政审批制度改革，简化程序，提高效率。全面推进政府信息公开，加强电子政务建设，办好政府网站和市长公开电话，增强政府工作的透明度，保障人民群众的知情权、参与权和监督权。自觉接受人大的法律监督和工作监督、政协的民主监督、新闻媒体的舆论监督和社会公众的全面监督，认真听取各民主党派、工商联、社会各界的意见和建议。深入开展“六五”普法活动，全面落实“五五”依法行政规划，不断提高依法行政能力。三是加强廉政建设。严格执行廉洁自律的各项规定。全面落实党风廉政建设责任制，按照“一岗双责”要求，加强对领导班子和领导干部的教育、管理和监督。完善源头治腐规章制度，大力推进行政审批、招投标平台建设，对行政审批和招投标工作实行全程监督。加大案件查办力度，严肃查办商业贿赂案件和工程建设、房地产开发、土地管理等领域的案件。深入开展行风政风评议活动，促进全市政府系统改善服务、提高效能。严厉打击公路和企业“三乱”行为，坚决纠正损害群众利益的不正之风，进一步优化经济发展环境。

　　各位代表，美好蓝图已经绘就，新的征程已经开启。光荣属于过去，奋斗成就未来。让我们在市委的坚强领导下，抢抓机遇、奋发进取，团结拼搏、扎实苦干，努力在打造鲁苏豫皖交界地区科学发展高地征程上创造更加辉煌的业绩！
